# Secondary Bile Acids and Short Chain Fatty Acids in the Colon: A Focus on Colonic Microbiome, Cell Proliferation, Inflammation, and Cancer

**DOI:** 10.3390/ijms20051214

**Published:** 2019-03-11

**Authors:** Huawei Zeng, Shahid Umar, Bret Rust, Darina Lazarova, Michael Bordonaro

**Affiliations:** 1 U. S. Department of Agriculture, Agricultural Research Service, Grand Forks Human Nutrition Research Center, Grand Forks, ND 58203, USA; bret.rust@ars.usda.gov; 2 Department of Surgery and University of Kansas Cancer Center, Kansas City, KS 66160, USA; sumar@kumc.edu; 3 Department of Medical Education, Geisinger Commonwealth School of Medicine, Scranton, PA 18509, USA; dlazarova@som.geisinger.edu (D.L.); mbordonaro@som.geisinger.edu (M.B.)

**Keywords:** bile acids, butyrate, colon cancer, microbiome, inflammation, obesity

## Abstract

Secondary bile acids (BAs) and short chain fatty acids (SCFAs), two major types of bacterial metabolites in the colon, cause opposing effects on colonic inflammation at chronically high physiological levels. Primary BAs play critical roles in cholesterol metabolism, lipid digestion, and host–microbe interaction. Although BAs are reabsorbed via enterohepatic circulation, primary BAs serve as substrates for bacterial biotransformation to secondary BAs in the colon. High-fat diets increase secondary BAs, such as deoxycholic acid (DCA) and lithocholic acid (LCA), which are risk factors for colonic inflammation and cancer. In contrast, increased dietary fiber intake is associated with anti-inflammatory and anticancer effects. These effects may be due to the increased production of the SCFAs acetate, propionate, and butyrate during dietary fiber fermentation in the colon. Elucidation of the molecular events by which secondary BAs and SCFAs regulate colonic cell proliferation and inflammation will lead to a better understanding of the anticancer potential of dietary fiber in the context of high-fat diet-related colon cancer. This article reviews the current knowledge concerning the effects of secondary BAs and SCFAs on the proliferation of colon epithelial cells, inflammation, cancer, and the associated microbiome.

## 1. Introduction

**Figure 1:**
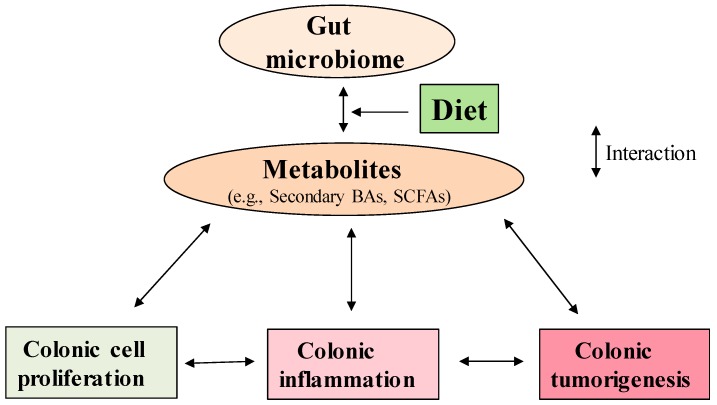
The interplay between gut microbiome, metabolites, colonic cell proliferation, inflammation, and cancer.

Colon cancer accounts for approximately 135,000 new cancer cases in the United States each year [1,2,3]. Diet-induced obesity is now established as a risk factor for cancer, and overweight and obesity affect two-thirds of Americans and an estimated 2.3 billion people worldwide [1,2,3]. The intake of saturated fatty acids (SFAs), mainly long-chain SFAs, is associated with obesity [4,5]. A “Western” diet that is high in fat (SFAs) and low in fiber promotes colonic inflammation and cancer [6,7]. Bile acid (BA) concentrations can reach 1 mM in the colon after the consumption of a high-fat meal [8,9], and these BAs, mostly secondary BAs in humans, are believed to be promoters of colon cancer [10,11]. Abnormally high secondary BA concentrations trigger a plethora of detrimental effects on the colonic mucosa, such as oxidative stress and inflammation [12]. Furthermore, research has shown that differences in the compositional nature of gut microbiome are associated with differences in risk of obesity and colon cancer via chronic inflammation [13]. In contrast, a growing body of evidence demonstrates that increased dietary fiber intake is associated with many beneficial effects including amelioration of obesity and anti-inflammatory and anticancer activities in the colon [10,14]. These effects might be due to the increased production of short-chain fatty acids (SCFAs), acetate, propionate, and butyrate, produced from dietary fiber through bacterial fermentation in the colon [7,10,14]. Consistent with these observations, a low intake (<15 g/day, a US adult) of dietary fiber not only leads to reduced microbial diversity and decreased SCFA production, but also shifts gut microbial metabolism toward the utilization of less favorable substrates, such as dietary fat, which increases BA production [15,16,17]. Subsequently, this process leads to an increase in potentially detrimental metabolites, such as secondary BAs, via biotransformation of BAs in the colon [15,16,17]. The cytotoxic and pro-inflammatory nature of these metabolites contributes to colonic inflammation and neoplastic development [15,16,17]. In view of the functional trade-off between gut bacterial metabolites from high-fat and high-fiber diets, a high-fiber diet is likely to counteract the detrimental effects of a high-fat diet although there are few studies in this regard [14,15,16]. This review focuses on the molecular effects of secondary BAs and SCFAs on colonic cell proliferation, inflammation, and colon cancer (Figure 1). 

## 2. Secondary BAs, SCFAs, and Microbiome

Humans and microbes have evolved symbiotically, and the human gut harbors trillions of microbes which are essential for host development and physiological function. The gut microbiome comprises bacteria, archaea, viruses, fungi, etc., which interact with each other and with the host to modulate host metabolism. The human gastrointestinal (GI) tract represents an abundant reservoir of microbes with over 100 trillion bacteria grouped in up to 1000 species [18,19]. Bacterial diversity in the gut is relatively low, as compared to microbial communities (>50 bacterial phyla) found in water and soil environments [7]. The bacterial constituents of human gut microbiota are dominated by two major phyla, *Bacteroidetes* and *Firmicutes*; whereas the phyla *Proteobacteria*, *Verrucomicrobia*, *Actinobacteria*, *Fusobacteria*, and *Cyanobacteria* are present in minor proportions [7,20]. Prevalent genera include *Bacteroides, Eubacterium, Bifidobacterium, Ruminococcus, Peptostreptococcus, Propionibacterium, Clostridium, Lactobacillus, Escherichia, Streptococcus*, and archaeal genus *Methanobrevibacter* [7,20,21]. The microbiome contributes to homeostatic regulation in many tissues in our body, and the interrelationship of hosts and their microbiota is a mutualistic symbiosis, which refers to a healthy balance of microbes in the gut [22,23]. However, once this mutualistic symbiosis is disrupted, it may lead to the development of chronic diseases including colonic inflammation and cancer [24].

### 2.1. Secondary BAs

BAs, normal metabolites in the intestinal lumen, are needed for digestion and absorption of lipids, as well as uptake of cholesterol and fat-soluble vitamins. In addition, BAs regulate intestinal epithelial homeostasis in the GI tract [25]. In the liver, primary BAs are conjugated to either glycine or taurine by the enzymes BA-CoA synthase (BACS) and BA-amino acid transferase (BAT) [25]. These conjugated BAs are subsequently stored in the gallbladder [25], and following cholecystokinin-stimulated secretion into the duodenum, contribute to the solubilization and digestion of ingested lipids through the small intestine and colon [25]. High-fat diets induce enhanced BA discharge resulting in increased colonic concentrations of primary BA compared with low or normal fat diets [25,26]. Conjugated primary BAs are reabsorbed in the distal ileum, primarily through active transport by the apical sodium-dependent bile salt transporter (ASBT) or the ileal BA transporter (IBAT) via enterohepatic circulation [25,27]. However, 5 to 10% of BAs that are not reabsorbed can serve as substrates for microbial metabolism and undergo biotransformation to secondary BAs, which may promote colon carcinogenesis [25,27]. The major biotransformations include: hydrolysis of conjugated BAs to free BAs and glycine or taurine by bile salt hydrolase (BSH); 7α-dehydroxylation of cholic acid (CA), and chenodeoxycholic acid (CDCA) yielding deoxycholic acid (DCA) and lithocholic acid (LCA), respectively; BA 7β-dehydroxylation of ursodeoxycholic acid (UDCA) yielding LCA [28]. The composition of bile salts in the small intestine is similar to the biliary pool; whereas, the BA profile in the colon is mainly unconjugated along with secondary BAs due to the action of bile salt hydrolases (BSH) and 7α-dehydroxylation [27]. Most BSH bacteria are Gram-positive gut bacteria including *Clostridium*, *Enterococcus*, *Bifidobacterium*, and *Lactobacillus*; whereas, members of the genus *Bacteroides* are the only Gram-negative bacteria with BSH activity [27,28]. Certain species of human intestinal archaea, such as *Methanobrevibacter smithii* and *Methanosphera stadmanae,* have been shown to encode BSH capable of hydrolyzing both taurine- and glycine-conjugates [27,28]. Importantly, BAs also change the structure of the gut microbial community because there is a dynamic interplay between host BAs and the microbial population in the gut. For example, feeding of cholic acids at mM levels (similar to the outcome of high-fat consumption) to rats significantly altered the microbiota at the phylum level, which resulted in an increase in *Firmicutes* and a reduction in *Bacteroidetes* [29]. In another study, a diet high in saturated milk-derived fats increased taurine-conjugated BAs, promoting the outgrowth of potentially pathogenic bacteria in the gut [30]. Thus, colonic BAs clearly play a major role in the composition of gut microbiome.

### 2.2. SCFAs

Dietary fiber constitutes a spectrum of non-digestible food components including non-starch polysaccharides, oligosaccharides, lignin, and analogous polysaccharides with associated health benefits [31,32]. The gut microbiota produces SCFA from fermentable non-digestible carbohydrate. An equation outlining overall carbohydrate fermentation in the colon was previously described [33]: 59C_6_H_12_O_6_ + 38H_2_O → 60acetate + 22propionate + 18butyrate + 96CO_2_ + 256H^+^. The total concentration of SCFAs in colonic contents may exceed 100 mM [34,35]. Acetate makes up ~60% to 75% of the total SCFAs, and is generated by many bacterial groups via reductive acetogenesis [36]. Acetate is produced from pyruvate via acetyl-CoA and via the Wood-Ljungdahl pathway [37,38]. The main acetate-producing bacteria are *Akkermansia muciniphila*, *Bacteroides* spp., *Bifidobacterium* spp., *Prevotella* spp., *Ruminococcus* spp., *Blautia hydrogenotrophica*, *Clostridium* spp., *Streptococcus* spp. [37,39]. However, the bacterial groups that generate propionate and butyrate (with important health benefits) are specialized. Propionate is synthesized from phosphoenolpyruvate (PEP) through the succinate pathway or the acrylate pathway, in which lactate is reduced to propionate [38]. The main propionate-producing bacteria are *Bacteroides* spp., *phascolarctobacterium succinatutens*, *Dialister* spp., *Veillonella* spp., *Megasphaera elsdenii*, *Coprococcus catus*, *Salmonella* spp., *Roseburia inulinivorans*, *Ruminococcus obeum* [37,40]. Butyrate is formed from two molecules of acetyl-CoA, yielding acetoacetyl-CoA, which is further converted to butyryl-CoA via β-hydroxybutyryl-CoA and crotonyl-CoA [38]. The main butyrate-producing bacteria are *Coprococcus comes*, *Coprococcus eutactus*, *Anaerostipes* spp., *Coprococcus catus*, *Eubacterium rectale*, *Eubacterium hallii*, *Faecalibacterium prausnitzii*, *Roseburia* spp. [37,40,41]. Collectively, there are different phylogenetic groups of bacteria responsible for acetate, propionate, and butyrate production. Therefore, this may allow for differential manipulation of their production by the gut microbiota.

Recent animal studies clearly demonstrate that dietary fiber not only physically hinders the BA reabsorption and cholesterol uptake, but also alters the BA profile with lower circulating levels without excess excretion in the feces [42,43]. Given that dietary fiber alters BA (including secondary BA) composition, it may suggest an association between the shift in colonic microbiome and SCFAs. It will be of great interest to examine the potential crosstalk between BAs and SCFAs via altering colonic microbiome composition.

## 3. The Impact of Secondary BAs and SCFAs on Colonic Cell Proliferation

Normal tissues tightly regulate mitogenic signals to ensure a homeostasis of cell numbers, which is critical for maintaining normal tissue architecture and function [44]. In contrast, the most basic feature of cancer cells is the ability to sustain chronic proliferation, and states of abnormally increased colonic epithelial cell proliferation are associated with the development of colon cancer in human and animal studies [44,45,46]. Lower rates of colon cancer cell proliferation were reported in vegetarians and in those following similarly health-conscious diets compared to individuals with a high risk of colon cancer [45,47]. However, the “western” diet that is high in fat and low in fiber, deregulates BA homeostasis and leads to an increase in colon cell proliferation in animal studies [48,49]. As such, it is essential to examine the effects of secondary BAs and SCFAs on colonic cell proliferation.

### 3.1. Secondary BAs and Colonic Cell Proliferation

Human population-based studies, including studies of colon cancer patients, have shown that high-fat diets exhibit elevated levels of fecal secondary BAs, mostly DCA and LCA [50,51,52]. Similarly, numerous mechanistic studies demonstrate that high-fat diets stimulate bile discharge and increase the concentration of BAs in the colon in animal models [25,26]. Secondary BAs exert detrimental effects on colonic epithelium architecture and function through multiple mechanisms, such as receptor-dependent signaling pathways, in cultured cells and animal models [53]. Furthermore, BAs that function as tumor promoters have been tested using a wide variety of experimental settings, including feeding BAs before, in association with, or after carcinogen exposure in a mouse model [54].

In addition to exerting a range of physiological activities, such as digestion and absorption of nutrients, the intestinal epithelium is the first line of defense against noxious agents and pathogens. Cellular membrane-perturbing effects and resulting signaling cascades are generated by secondary BAs but not primary BAs [55]. At low concentrations, secondary BAs (e.g., DCA, 5 μM) induce COX-2 expression through a transactivation of the epidermal growth factor receptor (EGFR). Furthermore, secondary BAs activate β-catenin cell-signaling, extracellular signal-regulated kinases 1 and 2 (ERK1/2) signaling via activator protein 1 (AP1) and c-Myelocytomatosis (c-Myc) target pathways, stimulating colon cancer cell proliferation and invasiveness [56,57,58]. Consistent with this hyperproliferation, increased BA levels also activate the nuclear factor kappa B (NF-κB) pathway [58,59,60]. However, colonic BAs can reach concentrations of up to 1 mM in the colon following a high-fat meal and, in humans, these are mostly secondary BAs [8,9,12]. In contrast to the effect of low secondary BA concentrations, we and others have shown that secondary Bas, such as DCA, at physiological concentrations (0.05 to 0.3 mM), inhibit colonic cell proliferation to varying degrees based on the type of cell [10,61]. Specifically, DCA and LCA promote cell cycle arrest and apoptosis primarily through the generation of intracellular reactive oxygen species (ROS), genomic DNA breakage, activation of ERK1/2, caspase-3 and poly(ADP-ribose) polymerase (PARP), but decreased cyclin E concentrations [10,60,61]. Similarly, studies have shown that DCA suppresses p53 by stimulating the process of proteasome-mediated degradation of p53 in response to DNA damage [62]. Furthermore, DCA activates cellular signaling pathways that lead to selective resistance to apoptosis, angiogenesis (prostaglandin E2 through vascular endothelial growth factor), proliferation and oxidative stress [62,63,64]. Therefore, in response to repeated DNA damage due to the exposure of secondary BAs, the large number of cell generations in the colonic (and other gastrointestinal) epithelia may allow time for induction and selection of mutations leading to cancer in humans [62,63,64]. 

### 3.2. SCFAs and Colonic Cell Proliferation

Complex carbohydrates are metabolized by the colonic microbiota to oligosaccharides and then are fermented to SCFAs. This may mechanistically explain the observation that dietary fiber reduces the risk of colon cancer in human populations and animal models [7,65]. The concentration of SCFAs varies along the large intestine, with highest levels in the cecum and proximal colon, and lowest toward the distal colon [35]. Colonic luminal SCFA (acetate, propionate, and butyrate) concentrations can reach 100 mM and are enhanced by consuming fermentable fiber [35,66,67]. Although acetate, propionate, and butyrate are all metabolized to some extent by the epithelium to provide energy and decrease the pH of the colon, butyrate plays the most critical role in maintaining colonic health and moderating cell growth and differentiation [68,69]. Butyrate is selectively taken up by the colonic epithelium (via monocarboxylate transporter 1 and other transporters) and provides colonocytes with approximately 70% of their energy [70] and is required for energy homeostasis [71,72]; whereas, acetate and propionate are primarily transported to muscle and liver tissue, respectively [73]. Within a cell, butyrate concentrations determine their fates: At low concentration (<0.5 mM), butyrate meets the cell’s energy needs and stimulates proliferation of normal colonic cells; at concentrations exceeding its energy needs (depending on the cell type, range: 0.5–5 mM), butyrate acts as a histone deacetylase inhibitor (HDACi) [74]. In addition, butyrate induces cell cycle arrest and apoptosis in both a p53-dependent and -independent manner at physiologically relevant intracellular concentrations (0.5–5 mM) [74,75]. Several in vitro studies have demonstrated that butyrate inhibits HDAC and allows histone hyperacetylation which leads to the transcription of many genes including p21/Cip1, and cyclin D3 [76,77]. The induction of the cyclin-dependent kinase inhibitory protein p21/Cip1 accounts for cell arrest in the G1 phase of the cell cycle [76,77]. Furthermore, butyrate treatment increases the phosphorylation of ERK1/2, a survival signal in noncancerous cells; whereas, it decreases p-ERK1/2 in cancerous cells [76]. We and others have also reported that at 0.5 mM or higher concentration, butyrate inhibits the migration and invasion rate of cancer cells by increasing the expression of anti-metastatic genes (e.g., metalloproteinases) and inhibiting the activation of pro-metastatic genes (e.g., matrix metalloproteinases) [78,79].

## 4. The Role of Secondary BAs and SCFAs in Colonic Inflammation

Intestinal immune homeostasis is tightly regulated by crosstalk between gut bacteria, mucosal immune cells, and intestinal epithelial cells. Although inflammation is a normal biological response to stress involving a complex network of cells and their intracellular and extracellular stimuli, chronic inflammation is a persistent inflammatory response leading to prolonged infiltration of immune cells, tissue hyperplasia and destruction [80,81]. Malfunction of this immune homeostasis may induce chronic inflammation, one of the key mechanisms promoting and accelerating cancer development [82]. This process mainly involves immune cell infiltration, continuous activity of various cytokines, chemokines, increased reactive oxygen species, and activation of key signaling pathways [83]. Secondary BAs and SCFAs exhibit pro-inflammatory and anti-inflammatory properties, respectively.

### 4.1. Secondary BAs and Colonic Inflammation

Chronic exposure to high BA levels can induce inflammation and cancer [64,84] In response to elevated concentrations of BAs, a complex of intestinal nuclear receptor and membrane Takeda G protein-coupled BA receptor-1 (TGR5) signaling network is activated [85,86]. This signaling network consists of farnesoid X receptor (FXR), vitamin D receptor (VDR), xenobiotic receptors [pregnane X receptor (PXR) and constitutive androstane receptor (CAR), *N*-acylphosphatidylethanolamine specific-phosphopholipase D (NAPE-PLD) and TGR5 [53,86,87,88]. The activation of these receptors and lipid signaling molecules coordinates the control of BA uptake, detoxification, regulation of fatty acid ethanolamide biosynthesis, and enteric hormone secretion [53,86,87,88]. Secondary BAs (e.g., DCA and LCA) regulate lipid signaling pathways (e.g., NAPE-PLD) and the immune system, in part, through their receptors, such as TGR5, FXR, and PXR [88,89,90,91,92]. For example, macrophages are major regulators of cytokine production in the gastrointestinal tract, and their action is partly activated by the binding of secondary BAs with the TGR5 receptor [93]. Macrophage polarization (pro-inflammatory M1 or anti-inflammatory M2) determines whether activation of TGR5 stimulates either pro-inflammatory or anti-inflammatory responses [94]. Interestingly, TGR5 activation also induces a partial transformation from the M1 to the M2 phenotype producing an elevated IL-10:IL12 ratio, and IL-10 subsequently inhibits pro-inflammatory cytokines, such as TNF-α and IL-6 [94]. In line with TGR5 anti-inflammatory property, pattern recognition receptors present on innate immune cells, such as toll-like receptors (TLRs), recognize pathogen-associated molecular patterns and activate immunity [95]. A recent study has shown, for the first time, that TGR5 activation significantly attenuates the up-regulation of the TLR4-NF-κB pathway, and reduces liver inflammation in a mouse model [96]. Conceivably, TGR5 may also exert anti-inflammatory response by mediating the TLR4 pathway in the colon. At relatively low concentrations (e.g., <50 μM) in combination with certain immunological milieu, secondary BAs may exert anti-inflammatory actions in the colon by decreasing proinflammatory cytokine levels [97]. However, at high physiological concentrations, secondary BAs can cause oxidative/nitrosative stress, DNA damage, apoptosis, and mutations [50,98].

Hydrophobicity is major determinant of BA toxicity. The magnitude of BA hydrophobicity is UDCA < CA < CDCA < DCA < LCA. While regulating receptor-dependent pathways, secondary BAs exert detrimental effects on the architecture and function of the colonic epithelium through multiple mechanisms including oxidative damage to DNA, as well as inflammation and activation of NF-κB pathway [54,90]. The detergent property of DCA causes membrane perturbations resulting in activation of protein kinase C and a release of arachidonic acid [64,99]. Subsequently, arachidonic acid is converted by the enzymes cyclooxygenase 2 (COX-2) and lipoxygenase to certain pro-inflammatory and pro-angiogenic prostaglandin species, and reactive oxygen species which cause DNA damage and inhibit DNA repair enzyme activities [57,59].

The above process stimulates the production of pro-inflammatory cytokines, such as IL-6 and TNF-α, that promote inflammation and inactivate FXR [100,101]. As a consequence, the inactivation or mutation of FXR leads to the pro-inflammatory status in the colon [89,90]. For example, animal studies showed that FXR^−/−^ mice were more susceptible to trinitrobenzenesulfonic-acid induced colitis than wild type (WT) mice [102]. Similarly, compared with WT mice, dextran-sulfate-sodium induced colitis was more severe in PXR^−/−^ mice [103] although the action of these receptors (TGR5, FXR, PXR) may also be determined by cell types, tissue site, age, and immunological context [104,105]. 

### 4.2. SCFAs and Colonic Inflammation

Inflammation is an immediate response of the body to tissue injury caused by microbial infection and other noxious stimuli. However, inadequate resolution of inflammation and uncontrolled inflammatory process causes a state of chronic inflammation, which is a common etiologic factor for cancer [106]. The maintenance of the intestinal epithelial barrier is critical to reducing systemic inflammation as evidenced by the emerging association between obesity and increased intestinal permeability [7,107]. Recent studies have suggested that SCFAs enhance the expression of epithelial barrier-forming molecules and mucin production, which are partly mediated by 5′ adenosine monophosphate-activated protein kinase (AMPK) activation, HDACi activity and TLR4 pathway [108,109,110,111]. It is known that SCFAs activate several cell surface G-protein-coupled receptors (GPCRs), such as GPR43, GPR41, GPR109A, and Olfr78 [112,113,114]. Intestinal epithelial cells and certain myeloid cells, such as neutrophils and macrophages, express GPR43, GPR41, and GPR109A at variable levels [112,113,114]. However, T- and B-cells do not express these SCFA receptors [105], and SCFAs exert immunosuppressive effects in both innate and adaptive immunity [115]. Leukocytes are recruited and migrate from the bloodstream to inflamed tissues through a multistep process that involves expression and activation of several proteins, such as adhesion molecules and chemokines [116], and SCFAs modify this leukocyte recruitment [115]. For example, a wide variety of cytokines and other proinflammatory mediators contribute to both extrinsic and intrinsic pathways of inflammation, and macrophages are the major source of these inflammatory mediators [106,117]. The activated macrophages produce significant amounts of mediators, such as TNF-α, IFN-γ and IL-6, chemokines, and nitric oxide (NO) [75,106]. 

Consistent with anti-inflammatory effects, either dietary consumption of soluble fiber pectin as an SCFA-delivery system to the colon or an orally administered butyrate reduces neutrophil recruitment and inflammation in mouse models [117,118,119]. SCFAs also directly regulate the antigen-specific adaptive immunity mediated by T- and B-cells. For example, butyrate activates intestinal macrophages through GPR109A to induce IL-10-producing T cells [120]. While the SCFA receptor-dependent mechanism is well recognized, the HDAC inhibitory property of SCFAs is also important. Inhibition of HDAC by butyrate regulates the transcription of certain cytokine genes, such as IFN-γ, TNF-α, and the activity of the NF-κB signaling pathway [121,122,123]. Subsequently, SCFAs, mainly butyrate, reduce the LPS- and cytokine-stimulated production of pro-inflammatory mediators (e.g., TNF-α, IL-6, IFN-γ, and NO) while increasing the release of the anti-inflammatory cytokine IL-10 [75,123,124]. Furthermore, with strong HDAC inhibitory effects, butyrate not only increases B-cell production of antibodies but also promotes B-cell differentiation into plasma B-cells [125]. Similarly, butyrate also promotes the activation and generation of anti-inflammatory regulatory T cells in the gut through HDAC inhibitory activities [126,127]. Although butyrate generally exhibits anti-inflammatory effects in the colon, it may exert either anti-inflammatory or pro-inflammatory effects on T cell-mediated immune responses depending on concentration and immunological milieu [128,129].

## 5. Secondary BAs, SCFAs, Oncomicrobes, and Colon Cancer

The pathogenesis of colon cancer is a multistep process that requires the alteration of multiple genes and pathways. More than 80% of colon cancer cases harbor aberrant wingless/integrated (Wnt)/β-catenin signaling, p53 phosphoinositide 3-kinase (PI3K), and transforming growth factor β (TGFβ) pathways that regulate colon cancer progression through intracellular mechanism or interaction with tumor microenvironment and cancer stem cells [130,131]. Emerging evidence indicates that secondary BAs and SCFAs play opposing roles in the development and progression of colon cancer. Indeed, key interactions amongst diet, the microbial community and their metabolites seem to prime the colonic mucosa towards neoplasia and through the adenoma–carcinoma sequence in colon cancer.

### 5.1. Secondary BAs and Colon Cancer

Epidemiologic studies have shown that subjects who consume a high-fat diet produce elevated levels of fecal secondary BAs, mainly DCA and LCA, as do colon cancer patients [132,133]. The intestinal mucosa is constantly renewed, and one of the primary forces involved in this process is the canonical Wnt/β-catenin signaling pathway [134,135]. 

First, colonic carcinogenesis is a progressive process including a sequence of cell mutations during the progression from adenoma to carcinoma, some of which result in deregulation of Wnt and apoptotic signaling pathways [134,135]. Cellular responses to secondary BAs in colonic tumorigenesis include the activation of Wnt and NFκB signaling pathways, DNA oxidative damage and impaired mitotic activities which lead to colonic cell hyperproliferation and invasiveness [94,135]. Increased colonic DCA and LCA concentrations promote apoptosis mainly through the activation of intrinsic apoptotic pathways including mitochondrial oxidative stress, reactive oxygen species (ROS), cytochrome C, and cytosolic caspases [94,136]. Subsequently, certain colonic epithelial cells may become resistant to BA-induced apoptosis, and this cell-subpopulation has been linked to colon cancer development [10,136].

Second, in response to toxic concentrations of BAs, a complex nuclear receptor network consisting of FXR, VDR, PXR, and CAR, coordinates the control of BA uptake and detoxification. These receptors also regulate cell cycle, mitosis, proliferation, and apoptosis [53,137]. In 2003, it was discovered that FXR expression was absent in certain colon cancer cell lines [138]. Later studies on intestinal tumor and adjacent normal mucosa have shown that FXR expression is strongly decreased during the transition from normal to neoplastically transformed epithelium [139]. Moreover, compared with WT mice, FXR^−/−^ mice (FXR deficiency) exhibited an increase in colonic inflammation and cancer risk [53,102]. In agreement with these results, FXR activation in colon cancer cells suppresses colonic epithelium proliferation, and induces pro-apoptotic genes, such as p21 while repressing antiapoptotic genes (e.g., *Bcl-2*) [137,140]. Collectively, these data demonstrate that FXR deficiency (even without elevated secondary BA levels) increases susceptibility to intestinal tumorigenesis via the perturbation of cell apoptotic events and intestinal mucosal integrity.

Third, the deregulation of the enzymatic detoxification system could increase the risk of colon cancer from chronic consumption of high-fat meals [141,142]. Several lines of evidence suggest that FXR exerts tumor-suppressive effects by transcriptional induction of detoxifying enzymes that metabolize and mediate excretion of toxic BAs [143,144]. FXR activation induces BA detoxification enzymes, such as cytochrome p450 3A4/3a11 (CYP3A4/Cyp3a11), aldo-keto reductase 1 B7 (AKR1B7), and cytosolic sulfotransferase (SULT) [143,144]. For example, the AKR1B7 protein is abundant in the enterohepatic system, where it catalyzes the conversion of 3α-hydroxy-BAs to 3β-hydroxy-BAs that are less cytotoxic than 3α-hydroxy-BAs (generated by enteric bacteria) [145]. These results ascertained the critical role of FXR in regulating the detoxifying enzyme expression. 

### 5.2. SCFAs and Colon Cancer

The prevention of colon cancer has been linked to multiple dietary factors including vegetarian diets and other high-fiber diets. Fecal SCFAs, especially butyrate, have been linked to the prevention of colon cancer and are derived primarily from the fermentation of dietary fiber [14,146,147]. Among the SCFAs produced in the colonic lumen via the fermentation of dietary fiber, butyrate is of interest for colon cancer prevention because it functions as a potent HDACi and promotes cell cycle arrest, differentiation, and/or apoptosis of colon cancer cells at physiological concentrations [14,148].

Recent research has focused on the signaling pathways through which butyrate may exert anti-proliferative and pro-apoptotic effects in colon epithelial tissues. The TGF-β signaling pathway is involved in cell sensitization to pro-apoptotic events in colonocytes, and downregulation of *mothers against decapentaplegic homolog* 3 (*SMAD*3), a TGF-β regulatory gene, leads to cancer progression [149,150]. Butyrate treatment (in mM concentrations) induces *SMAD*3 mRNA in a time-dependent manner, and potentiates the effects of TGF-β signaling on growth inhibition in the gut [151]. In addition, butyrate treatment results in genomic DNA fragmentation, apoptosis, and an increase in G1/G2 phase cell cycle arrest in colon cancer cells [10,76]. Furthermore, we have recently demonstrated the differential roles of butyrate in cell proliferation and the activation of ERK1/2 signaling, histone hyperacetylation, c-Myc, p21 protein abundance and intracellular location in cancerous and noncancerous colon cells, which may account for butyrate’s selective anticancer-potential [76].

The mechanistic action of butyrate is implicated in the Wnt pathway that is mediated by an upregulation of active β-catenin, which can be associated with either of two histone acetyltransferases, the cAMP-response element-binding protein (CREB) binding protein (CBP) or p300 [148,152]. Wnt signaling mediated by CBP is associated with colonic cell proliferation, while Wnt signaling mediated by p300 is more closely associated with differentiation; thus, it is likely that CBP-Wnt activity is predominant in cancer [148,152]. Subsequently, hyperactivation of this pathway by butyrate (or by other HDACis) leads to enhanced transcription of Wnt signaling-related proteins involved in the apoptosis of colon cancer cells [148,152]. How are these findings consistent with the ability of Wnt signaling to promote proliferation in normal cells and the established contribution of mutation-deregulated Wnt signaling to uncontrolled proliferation in cancer? A solution to this conundrum is offered by the “just right hypothesis” for colon cancer development [153]. We have proposed that butyrate-induced hyperactivation of Wnt signaling takes place only in neoplastic colonic cells that exhibit mutation-deregulated Wnt pathway [153]. Such tumors constitute the large majority of sporadic colon cancer cases, as well as all cases of familial adenomatous polyposis [153]. In contrast, butyrate is a main energy source [70] in normal colonic epithelial cells, and supports cell proliferation by maintaining low levels of Wnt signaling [153]. These findings may explain the “butyrate paradox”, e.g., the differential effect of butyrate on normal and neoplastic colonic cells. In addition, this hyperactivation of Wnt signaling in neoplastic cells containing mutations that activate the pathway, differs from the Wnt signaling activation induced by BAs mentioned above. Thus, butyrate-induced Wnt signaling hyperactivation in neoplastic cells is expected to increase levels of Wnt activity to the range resulting in apoptosis; whereas, the stimulation of normal colonic cells by BAs is likely to result in moderate levels of Wnt activity that promote proliferation.

In addition to possessing activating mutations in the Wnt signaling pathway, cancer cells tend to have aberrant metabolism, with an increased reliance on aerobic glycolysis, rather than oxidative phosphorylation (e.g., “Warburg effect”). Therefore, neoplastic colonic cells may preferentially utilize glucose, rather than butyrate as an energy source, and allow more butyrate to function as a HDACi [154] that stimulates Wnt signaling and induces apoptosis. As stated above, the hyperactivation of Wnt signaling in butyrate-treated colon cancer cells is a required event to achieve a high level of apoptosis in these cells; whereas, moderate Wnt activity has been associated with cancer cell proliferation [155]. However, cancer cells may gradually become resistant to the effects of butyrate [152]. This resistance may indicate a transition from a β-catenin-dependent Wnt pathway to a modified pathway, which does not depend on β-catenin for its downstream effects [152,156]. Overall, our data demonstrate that butyrate hyperactivates Wnt signaling in colon cancer cell lines that exhibit deregulated Wnt signaling due to mutations, and such mutations are a common feature of most colon cancer types [148,152,153]. The hyperactivation of the pathway is causally associated with butyrate-induced apoptosis of colonic neoplastic cells, and this effect may in part explain the preventive role of fiber against colon cancer.

### 5.3. Oncomicrobes and Colon Cancer

In addition to an elevated level of secondary BAs, consumption of a high-fat diet not only increases inflammatory status but also accompanies an increase of opportunistic pathogenic bacteria in the mouse colon [49,157]. Colonic inflammation and cancer are multifactorial disorders that may be affected by the complex interactions between genetics, diet, the gut microbiome, and other factors [158,159]. The theory that bacterial infection directly triggers colon cancer remains poorly understood. However, a concept is emerging wherein, certain pro-oncogenic bacteria have the potential to promote neoplastic transformation of epithelial cells of the colon, thereby, acting as driver oncomicrobes [160]. For example, certain colonic *Escherichia coli* strains in humans harbor a genomic island which encodes the polyketide-peptide (genotoxin colibactin) [161,162]. Colibactin is able to penetrate the cell membrane of colonocytes and cause DNA damage in the host cells resulting in mutagenesis [161,162]. Subsequently, these pro-oncogenic bacteria can remodel the mucosal immune response and bacterial composition to initiate colon cancer development [161,163]. These findings are consistent with the α-bug hypothesis in which certain microbiome members with unique virulence traits-bacteria (α-bugs) not only are directly pro-oncogenic, but also alter the colonic microbiome composition resulting in colon cancer [159,164]. However, the progress of α-bug’s action is likely dependent on the microbiome and its metabolite profiles in the colon. It remains to be determined how secondary BAs and SCFAs may change the abundance of pro-oncogenic bacteria and their activities in the colon. Whether these microbes represent the potential targets of chemopreventive strategies remain to be elucidated.

## 6. Concluding Remarks

**Figure 2:**
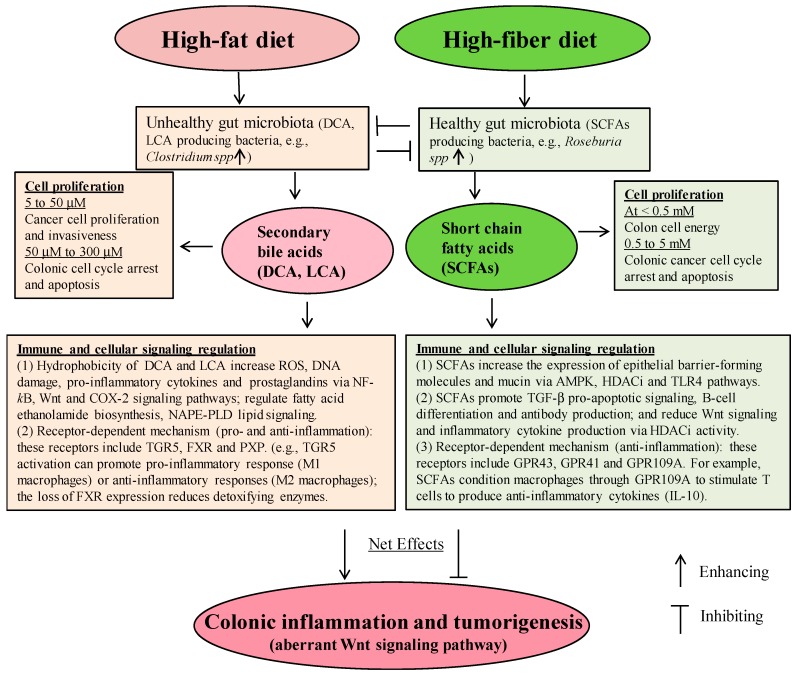
The proposed interaction of primarily functional pathways related to secondary bile acids (BAs) and short chain fatty acids (SCFAs) in the colon.

Secondary BAs and SCFAs are two major metabolites which play common and distinct roles in colonic cell proliferation (Figure 1). At low concentrations (e.g., <50 μM DCA and <0.5 mM butyrate) these microbial metabolites may promote colonic cell proliferation. In contrast, at high concentrations, secondary BAs and SCFAs (e.g., ~200 μM DCA and ~ 2 mM butyrate) inhibit colonic cell proliferation through common or differential molecular pathways (Figure 2). The crosstalk between BAs and SCFAs may occur through altering colonic microbiome composition (Figure 2). However, it remains to be determined which specific biochemical pathway(s) may be responsible for potential crosstalk between the signaling of BAs and that of SCFAs because their effects are not only based on concentration and duration but also on the type of cell. Secondary BAs and SCFAs regulate NAPE-PLD lipid signaling, the immune system and cellular signaling pathways with or without a direct activation of host receptors, including TGR5, FXR, PXR, GPR109A, GPR43, and GPR41 in the immune cells (Figure 2). At chronically high physiological levels, secondary BAs and SCFAs cause opposing effects on colonic inflammation and cancer. Because gut dysbiosis alters the production of secondary BAs and SCFAs, and leads to colonic inflammation, balancing nutrient intake with high fiber and low saturated fat intake is critical for maintaining a healthy gut microbiome. Conceivably, more information is needed to understand the immune regulatory function of colonic metabolites at the individual metabolite-species level and their downstream molecular processes. More importantly, it remains to be investigated how secondary BAs and SCFAs in combination may regulate colonic inflammation. A greater understanding of the action of these microbial metabolites may open new avenues for seeking noninvasive inflammatory biomarkers.
